# Porous La-Fe-O Perovskite as Catalyst for Combustion of Volatile Organic Compounds

**DOI:** 10.3390/ma18092008

**Published:** 2025-04-29

**Authors:** Corneliu Doroftei, Gabriel Murariu, Marius Dobromir

**Affiliations:** 1 Science Research Department, Institute of Interdisciplinary Research, Research Center in Environmental Sciences for the North-Eastern Romanian Region (CERNESIM), Alexandru Ioan Cuza University of Iasi, Bulevardul Carol I, Nr. 11, 700506 Iasi, Romania; 2 Physics, Chemistry and Environment Department, Faculty of Sciences and Environment, ‘’Dunarea de Jos’’ University of Galati, 111 Street Domneasca, 800201 Galati, Romania; 3 Science Research Department, Institute of Interdisciplinary Research, Research Center on Advanced Materials and Technologies (RAMTECH), Alexandru Ioan Cuza University of Iasi, Bulevardul Carol I, Nr. 11, 700506 Iasi, Romania

**Keywords:** lanthanum perovskite, LaFeO_3_, sol–gel self-combustion, structural properties, catalytic combustion, volatile organic compounds

## Abstract

Porous nanocrystalline lanthanum perovskite La-Fe-O (LaFeO_3_) powders were synthesized by the sol–gel self-combustion method, using polyvinyl alcohol as the colloidal medium. The perovskite structure of the material, without secondary phases, was obtained at a calcination temperature of 900 °C for 40 min. The obtained powder was tested for catalytic activity at moderate temperatures (50–550 °C) for ethanol, methanol, acetone, benzene, and Pb-free gasoline vapors. Catalytic combustion begins at quite low temperatures (60–200 °C), compared to normal combustion, and this can be attributed to the nanometric crystallites, the large specific surface area, and the presence of iron cations with different valences, Fe^3+^/Fe^2+^, resulting from the method we used to obtain the material. The degree of conversion reaches values of over 99% for acetone and ethanol vapors at a temperature of 270 °C and 310 °C, respectively, and over 97% for methanol vapors at a temperature of 330 °C. The degree of conversion for Pb-free gasoline and benzene reaches somewhat lower values, over 88% at much higher temperatures, 470 °C and 550 °C, respectively. The lanthanum perovskite catalyst, LaFeO_3_, obtained by the presented preparation method, can be recommended for the combustion of acetone, ethanol, and methanol vapors. The performance of this catalyst is remarkable and can be compared to that of a catalyst containing noble metals in its composition.

## 1. Introduction

The most unique properties of ceramic materials are their relatively high thermal, chemical, and mechanical stability [1,2,3]. Among these, the oxide compounds with the perovskite-type structure are at the forefront of research because they present structural, electrical, optical, magnetic, transport, and catalytic properties of great theoretical and practical importance. The oxide compounds with the perovskite-type structure have the general chemical formula XYO_3_ like the “perovskite” mineral CaTiO_3_, which has a face-centered cubic crystal structure, but they can have other crystal structures. The unit cell of a perovskite with a cubic structure consists of a single molecule. At the vertices of the cube are the X ions with a larger ionic radius, at the center of the cube are the Y ions with a smaller ionic radius, and the O ions are found in the centers of the cube faces. Perovskites generally have an alkali metal or rare earth cation at the X position and a transition metal cation at the Y position. The X cation generally has a larger ionic radius than the Y cation. Catalytic combustion at low and medium temperature is used to eliminate from air the polluting gases and volatile organic compounds (VOC), such as solvents vapors, mine gases, combustible gases released from industrial installations, etc. Generally, it is used when the concentration of combustible gases in air is very reduced and unable to keep the fire alive [4]. Still, another possible utilization now studied is for fuel cells that can work at such temperatures [5,6,7].

A good catalyst for catalytic combustion of gases or vapors must satisfy four major conditions: reduce the combustible gas concentration to minimum (I); activate at minimum values of gas concentration (II); manifest catalytic conversion at minimum temperature (III); and have a maximum utilization duration until deactivation (poisoning) (IV) [5].

The catalytic activity of oxide compounds with a perovskite-type structure in the oxidation of combustible gases and vapors is essentially controlled by the cation at the Y position [8,9,10,11]. Many studies have suggested that oxygen vacancies in perovskite-type oxide compounds play a major role in catalytic oxidations, although the origin of their catalytic activity is still debated [12,13,14,15]. In a XYO_3_ perovskite, the presence of different valences of the X or Y cations generates oxygen vacancies, which are preferential sites for O_2_ adsorption [8,11]. Important for the activity of perovskites as catalysts in the flameless catalytic combustion of volatile organic compounds (VOCs) is the mobility of oxygen [16,17,18]. The mobility of oxygen within the perovskite lattice is affected by the nature of the cation at position X and increases proportionally to the concentration of oxygen vacancies [6,8,19].

Lanthanum perovskite La-Fe-O (LaFeO_3_) is an oxide compound, and it has been extensively studied with reference to several potential applications, such as gas sensors, humidity sensors, and, last but not least, catalysts [20,21]. This oxide material usually crystallizes in a cubic or orthorhombic perovskite structure [21]. LaFeO_3_ perovskites usually exhibit structural defects due to fluctuations between two stable iron oxidation states generating oxygen vacancies.

Pecchi et al. [22] have undertaken a series of investigations on the catalytic activity of LaFeO_3_ perovskite in methane (CH_4_) combustion. The perovskite was obtained by the coprecipitation method, using sodium hydroxide as the precipitating agent, and sintering was carried out at a temperature of 973 K in air for 6 h. They obtained a 50% conversion for temperatures between 558 and 583 °C.

Also, Liu et al. [23] studied the catalytic activity of La-Fe oxide mixtures in methane combustion. They used the citrate method to prepare the compounds, using citric acid as a chelating agent, and sintering was carried out at a temperature of 973 K in air for 5 h. Regarding methane conversion, they obtained results comparable to those presented in the previous study.

Another study, conducted by Saberi et al. [24], presents the sensing and catalytic activity of palladium-doped LaFeO_3_ perovskite for propane (C_3_H_8_) and carbon monoxide (CO). The compound was obtained via the sol–gel combustion method, using sorbitol (C_6_H_14_O_6_) as colloidal medium. After combustion, the powder was calcined at a temperature of 600 °C in air for 3 h and then impregnated with an adequate amount of H_2_PtCl_6_ by the dry impregnation method to obtain 1.0% by weight of Pt/LaFeO_3_. It was subsequently calcined again at 600 °C in air for 3 h. The compound shows a conversion degree of carbon monoxide of 50% at 260 °C and 90% at 290 °C. Regarding propane, the compound shows a conversion degree of 50% at a temperature of 290 °C and 90% at a temperature of 360 °C.

Generally, nanostructured oxide compounds [4,25,26,27,28] obtained by various procedures [25,26,29,30] are used. The nanostructure is the one which provides a large specific surface area and a superior catalyst reactivity [30]. Particles’ shape, dimension, and superficial structure are determined by their preparation procedure. There are numerous procedures to produce nanoparticles of oxide compounds, some general and others specific to catalysts. Preparation procedures for nanoparticles with catalytic properties represent the object of numerous research studies and invention patents.

In this work, we synthesized the catalytic material (lanthanum perovskite, LaFeO_3_), in the form of a porous nanocrystalline powder via the sol–gel self-combustion method, using polyvinyl alcohol (PVA) as colloidal medium. The rapid heating and cooling during the autocombustion reaction produce nanopowders with reproducible properties, high specific surface area, and high effective porosity, which is beneficial for catalytic activity. This method is superior to other known conventional methods [31]. The obtained powder was investigated in terms of structural properties and catalytic activity at moderate temperatures (50–550 °C) for air-diluted vapors of ethanol, methanol, acetone, benzene, and Pb-free gasoline.

## 2. Materials and Methods

Nanocrystalline LaFeO_3_ perovskite was prepared by the sol–gel self-combustion method, using PVA as colloidal medium. A gel was obtained using stoichiometric amounts of metal nitrates dissolved in distilled water. A solution of PVA (10%) was mixed with the nitrate solution in a mass ratio of 1/1, resulting in a colloidal medium. With the help of a 10% ammonium hydroxide solution, the pH of the colloidal solution was brought to the value of 8, obtaining a sol of metal hydroxides, in accordance with the following equations:

La(NO_3_)_3_ + 3·NH_4_OH → La(OH)_3_ + 3·NH_4_NO_3_ (1)

Fe(NO_3_)_3_ + 3·NH_4_OH → Fe(OH)_3_ + 3·NH_4_NO_3_ (2)

The soil obtained was mixed at 80 °C, dried at 120 °C, and finally ignited, with an exothermic combustion reaction taking place:

C_2_H_3_OH + 5·NH_4_NO_3_→ 2·CO_2_ ↑ + 12·H_2_O ↑ + 5·N_2_ ↑ + Q (3)

As a result of the spontaneous combustion reaction, the dry soil turns into a powder. The burning reaction of the soil converts metal hydroxides into metal oxides, with the latter ones fusing in a solid state forming the perovskite structure, according to the following equations:

2·La(OH)_3_ + 2·Fe(OH)_3_ → La_2_O_3_ + Fe_2_O_3_ + 6·H_2_O (4)

La_2_O_3_ + Fe_2_O_3_ → 2·LaFeO_3_ (5)

Afterwards, the powder was calcined at a temperature of 500 °C for 30 min to remove traces of carbon and then was subjected to a heat treatment in air at a temperature of 900 °C for 40 min, resulting in the perovskite structure.

The transition temperatures during the formation process of perovskite-type phases for powders obtained by self-combustion were determined from thermogravimetric measurements and differential thermal analysis (TG–DTA) in the temperature range of 25–1000 °C, at a heating rate of 10 °C/min in static air. The crystal structure and phase formation of the powders were analyzed by X-ray diffraction (XRD) measurements at room temperature, in the 2*θ* range, between 20° and 80°, using CuKα radiation (λ = 1.541820 Å). The morphological and elemental surface structure was analyzed by scanning electron microscopy and energy-dispersive X-ray spectroscopy (SEM-EDX). The specific surface area, S_BET_, of the studied powders was obtained from the N_2_ adsorption–desorption isotherms at 77 K, using the BET (Brunauer, Emmet, and Teller) method. Also, the pore size distribution (PSD) curves were determined using the BJH (Barret, Joygner, and Halenda) method [32]. The determination of the chemical valence states present on the surface was achieved through X-ray photoelectron spectroscopy (XPS), using AlKα radiation (hν = 1486.6 eV).

The powder was studied from the point of view of catalytic activity (catalytic combustion) in the temperature range of 50–550 °C for a series of volatile organic vapors (VOCs) diluted with air, ethanol (C_2_H_5_OH), methanol (CH_3_OH), acetone (CH_3_COCH_3_), benzene (C_6_H_6_), and Pb-free gasoline, using a flow-type setup previously described in Ref. [33]. The heat-treated powder, representing the catalyst material, was placed in a tubular quartz reactor with a diameter of 8 mm, with automatic temperature regulation. A quantity of 0.5 g of catalyst material was used in the form of powder, and the gas entered the reactor passing through the entire volume of the powder at a pressure close to atmospheric pressure. The gas flow rate used was 100 cm^3^/min, with a concentration of 1–2‰ in air and a gas hourly space velocity (GHSV) of 5200 h^−1^. The catalytic activity of VOCs over the studied LaFeO_3_ powders was evaluated by the degree of conversion, C, of the gas passed through the reactor, expressed by the following relation [34]:

*C* = (*c_in_* − *c_out_*)/*c_in_*·100 (%), (6)

where *c_in_* and *c_ou_*_t_ are the inlet and outlet gas concentration, respectively. The concentrations were measured by a detection module with photoionization (PID-TECH) for gases and vapors. The catalytic tests were carried out both when the temperature increased and when the temperature decreased. Also, the tests were repeated over time, obtaining similar results, thus indicating the stability of the catalyst without its deactivation.

## 3. Results and Discussions

### 3.1. Structural and Morphological Characterization

**Figure 1:**
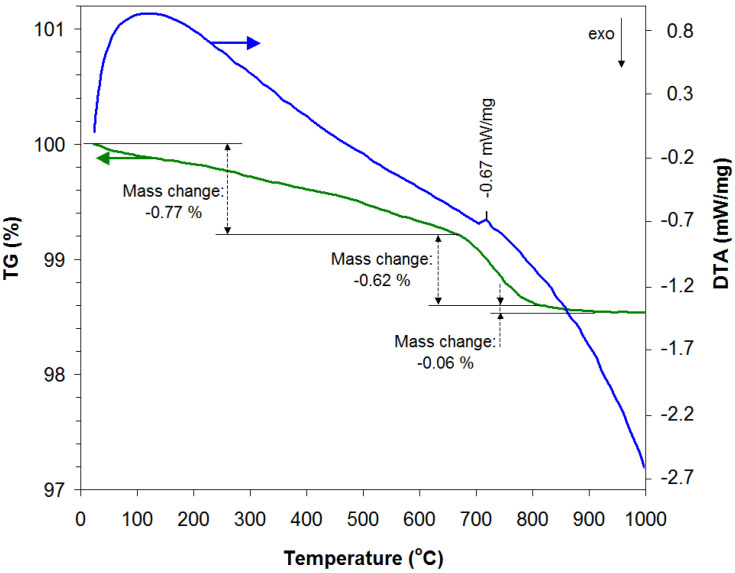
TG-DTA analyses for lanthanum perovskite, La-Fe-O.

In order to obtain a good crystallinity of the material without significantly affecting the nanodimensional properties, the accentuated porosity that the perovskites generally have by the method used by us (sol–gel self-combustion method using PVA as colloidal medium), the optimal thermal treatment temperature of the powder was determined by thermogravimetric analysis (by thermogravimetric analysis) and differential thermal analysis (TG-DTA). Figure 1 shows the TG-DTA analysis of the powder obtained following the self-combustion process and not thermally treated. From the results obtained from this analysis, it is noted that the mass of the powder generally decreases uniformly (−0.77%) with increasing temperature up to approximately 700 °C as a result of the loss of volatile substances, traces of residual carbon, and possible crystallization reactions. Corresponding to the temperature of 720 °C, the DTA curve indicates the presence of an endothermic peak, while the TG curve shows a sudden decrease in the powder mass (−0.62%) up to temperatures around 800 °C, which can be interpreted by the occurrence of crystallization reactions, indicating the appearance of crystalline phases of lanthanum perovskite. For temperatures between 800 and 900 °C, the powder mass shows a slight decrease (−0.06%), tending to remain constant for temperature values greater than or equal to 900 °C, which can be interpreted as the completion of the formation of the crystalline structure of this perovskite. Considering the results obtained from this analysis, the powder obtained from self-combustion was calcined at 500 °C for 30 min to burn residual carbon and any remaining organic substances, and, subsequently, it was heat treated in air at 900 °C for 40 min.

**Figure 2:**
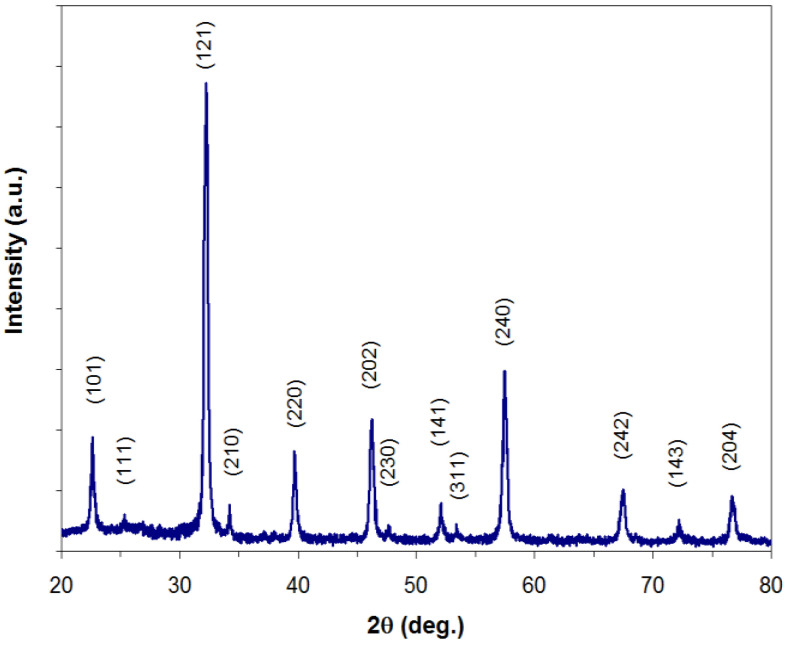
XRD patterns of lanthanum perovskite La-Fe-O heat-treated in air at 900 °C for 40 min.

Figure 2 shows the X-ray diffraction patterns (CuKα radiation source, wavelength λ = 1.541820 Å) of lanthanum perovskite La-Fe-O heat-treated in air at a temperature of 900 °C for a time of 40 min. From the XRD analysis, it is evident that the lanthanum perovskite, LaFeO_3_, was obtained with good crystallinity, without the presence of secondary phases (according to the PDF card Nos. 37–1493 for the lanthanum perovskite, LaFeO_3_), thus confirming the values of the powder heat treatment parameters, established as a result of the interpretations of the TG-DTA analyses. The compound exhibits the orthorhombic symmetry (space group Pnma), and the values obtained for the lattice parameters, a = 5.567 Å, b = 7.864 Å, and c = 5.556 Å, are close to the values obtained by other authors [35,36,37], who prepared the lanthanum perovskite, LaFeO_3_, by different methods.

**Figure 3:**
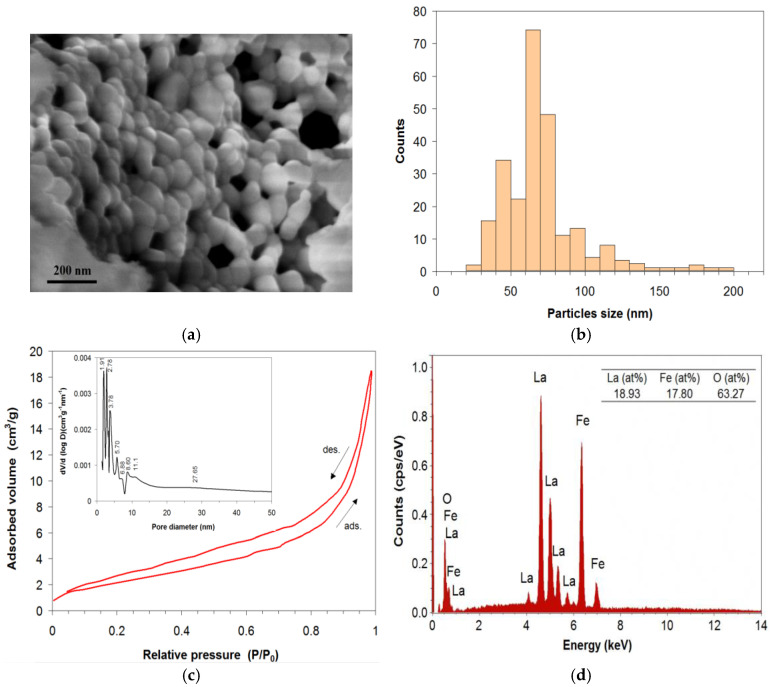
SEM micrograph (**a**), particles size distribution histogram (**b**); N_2_ adsorption–desorption isotherms and pore size distribution curve (inserted figure); (**c**) and EDX spectrum with the analyzed elements of (**d**) lanthanum perovskite La-Fe-O heat-treated in air at 900 °C for 40 min.

Figure 3a shows the SEM micrograph of lanthanum perovskite heat-treated in air at 900 °C for 40 min. Generally, the obtained perovskite is characterized by a fine granular structure, with grain sizes varying between 28 nm and 200 nm and the average grain size having a value of 69.28 nm. Nanoparticles form agglomerations of sizes between 100 nm and 300 nm, with predominating open pores that are distributed along the particle agglomerations. A particles size distribution histogram determined from the SEM micrograph is presented in Figure 3b. From the analyses of the N_2_ adsorption–desorption isotherms (Figure 3c) using the BET method [32], it results that the studied perovskite has a specific surface area, S_BET_, with a value of 8.48 cm^2^/g. The pore size distribution curve obtained from N_2_ desorption isotherm by the BJH method [32] is inserted in Figure 3c. The pore sizes (2–40 nm) fall within mesoporous region (2–50 nm) [32], and the total pore volume has a value of 0.0185 cm^3^/g. Since heterogeneous catalysis is a surface phenomenon, its efficiency is determined both by the chemical composition and the structure of the catalyst surface. A nanometric structure ensures a large specific surface area and a superior reactivity of the catalyst [30,38].

The surface elemental composition and homogeneity of the perovskite obtained were studied via energy dispersive X-ray (EDX) analysis. The EDX spectra (Figure 3d) evidenced the presence of La, Fe, and O elements and no other impurity element. From the elemental composition values, it can be deduced that the obtained lanthanum perovskite has a chemical composition very close to the nominal one; that is, the M/Mt ratios (M is the amount of a metal in the metal composition, and Mt is the total amount of metals on the surface) [39] are very close to the value 0.5, which represents the theoretical value. The La(at%)/[La(at%) + Fe(at%)] or Fe(at%)/[La(at%) + Fe(at%)] ratio is close to 0.5. This proves that the elements are uniformly distributed on the surface structure of the perovskite, and this result is largely due to the preparation method used—sol–gel self-combustion method.

**Figure 4:**
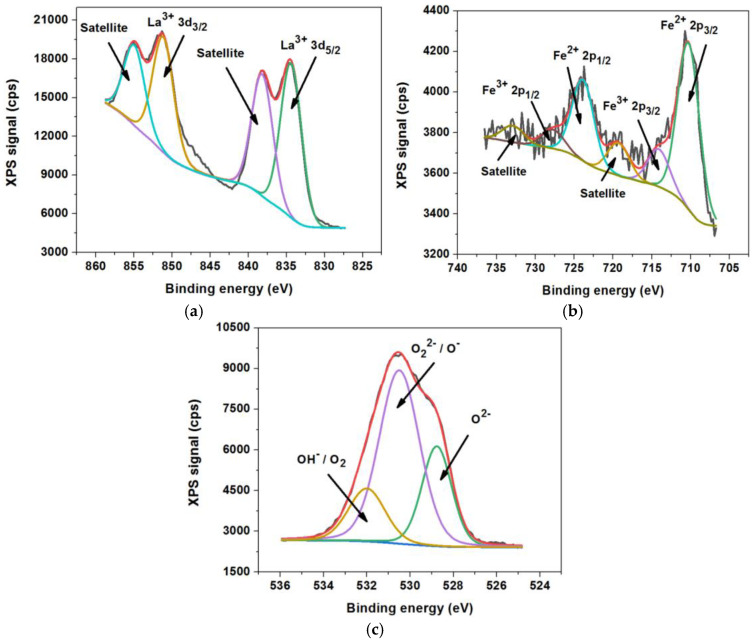
XPS spectra assigned to La 3d (**a**), Fe 2p (**b**), and O 1s (**c**).

X-ray photoelectron spectroscopy (XPS) was used to analyze the chemical valence states on the surface of the catalytic oxide compound, as well as the presence of oxygen vacancies that have an important role in the catalytic activity, being preferential points for O_2_ adsorption [8,9,10,11,12,13,14,15]. Figure 4 shows the XPS spectra assigned to La 3d, Fe 2p, and O 1s, as well as their deconvolutions.

The high-resolution spectrum assigned to La 3d (Figure 4a) presents the typical characteristics of La^3+^ ions, with two double peaks representing the spin–orbit splitting components of 3d_5/2_ (located at 834.50 eV and 838.30 eV) and 3d_3/2_ (located at 851.33 eV and 855.14 eV), respectively [40,41,42].

The high-resolution spectrum assigned to Fe 2p (Figure 4b) is represented by the binding energies of Fe 2p_3/2_ at 710.75 eV and Fe 2p_1/2_ at 723.87 eV, preceded by satellite peaks located at 719.42 eV and 733.16 eV, respectively. From the analysis of the deconvolution curves of the spectrum, the presence of Fe^3+^ cations was confirmed through the satellite deconvolution peaks located at 719.42 eV and at 733.16 eV, as well as through the main deconvolution peaks located at 714.38 eV and at 728.07 eV. From the analysis of the deconvolution curves of the spectrum, we also confirmed the presence of Fe^2+^ cations through the main deconvolution peaks located at 710.31 eV and 723.87 eV [41,43,44].

The high-resolution spectrum assigned to O 1s (Figure 4c) is represented by an asymmetric peak (located at 530.57 eV) characteristic of oxide compounds containing transition metals, also indicating the presence of Fe^2+^ cations that favor the generation of oxygen vacancies [38,45]. From the analysis of the deconvolution curves of the O 1s spectrum, we confirmed the presence in the lattice of several oxygen species, O^2−^, O_2_^2−^/O^−^, and OH^−^/O_2_, attributed to the peaks of the deconvolution curves located at 528.76 eV, 530.50 eV, and 532.07 eV, respectively [46].

### 3.2. Catalytic Activity

**Figure 5:**
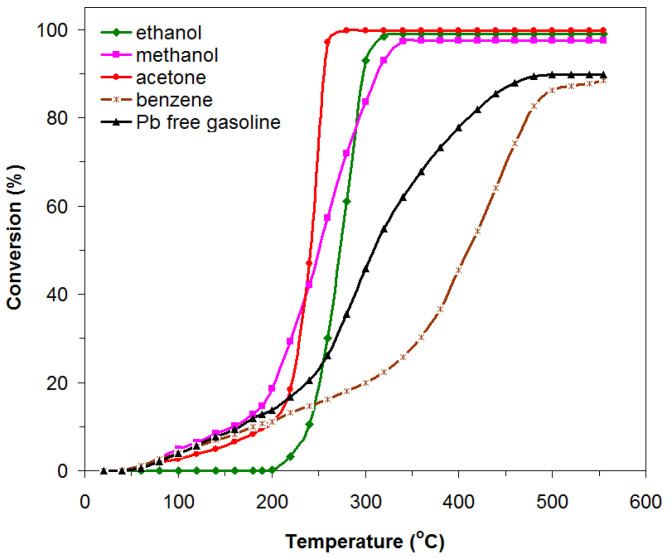
Conversion of ethanol, methanol, acetone, benzene, and Pb-free gasoline as a function of the operating temperature of the LaFeO_3_ catalyst (1–2‰ ethanol in air, 0.5 g catalyst, and GHSV = 5200 h^−1^).

The heat-treated powder, representing the catalyst material, was tested in terms of catalytic activity (catalytic combustion) in the temperature range of 50–550 °C for a series of alcohols, ketones, and hydrocarbon vapors (ethanol, methanol, acetone, benzene, and Pb-free gasoline) diluted in air. The degree of conversion, C, of the studied gas as a function of the reaction temperature for catalytic flameless combustion is shown in Figure 5. As can be seen in Figure 5, catalytic conversion begins at quite low temperatures (60–200 °C) compared to the normal combustion temperature, and this can be attributed to a large extent to the method we used to obtain the material, leading to obtaining nanometric crystallite sizes, an increased specific surface area, and the presence of iron cations with different valences, Fe^3+^/Fe^2+^. It is also worth noting that the temperature at which the conversion of ethanol vapors begins is higher (200 °C) than the temperature at which the conversion of the other vapors studied begins, namely methanol, acetone, benzene, and Pb-free gasoline (about 60 °C). This is due to the fact that ethanol decomposes at certain temperatures in the presence of the catalyst, into a series of intermediate products (ethylene, acetaldehyde, CO_2_, and other oxygenic compounds) [47,48], thus leading to an increase in the temperature value at which the conversion begins. The degree of conversion as a function of the reaction temperature is represented by specific S-shaped curves, indicating that the catalytic activity of the studied lanthanum perovskite is influenced to a greater extent by the reaction temperature in the case of alcohols and ketones than in the case of hydrocarbons.

In the case of ethanol vapors, the conversion starts around the temperature 200 °C and increases in the temperature range of 200–310 °C, reaching a value of over 99%, a value that is maintained as the temperature continues to rise. Close values regarding the degree of conversion are also obtained in the case of acetone (99%) and methanol (97%) vapors, at temperatures around 270 °C and 330 °C, respectively. The degree of conversion for Pb-free gasoline and benzene vapors reaches somewhat lower values, around 88% at much higher temperatures, namely 470 °C and 550 °C, respectively.

**Figure 6:**
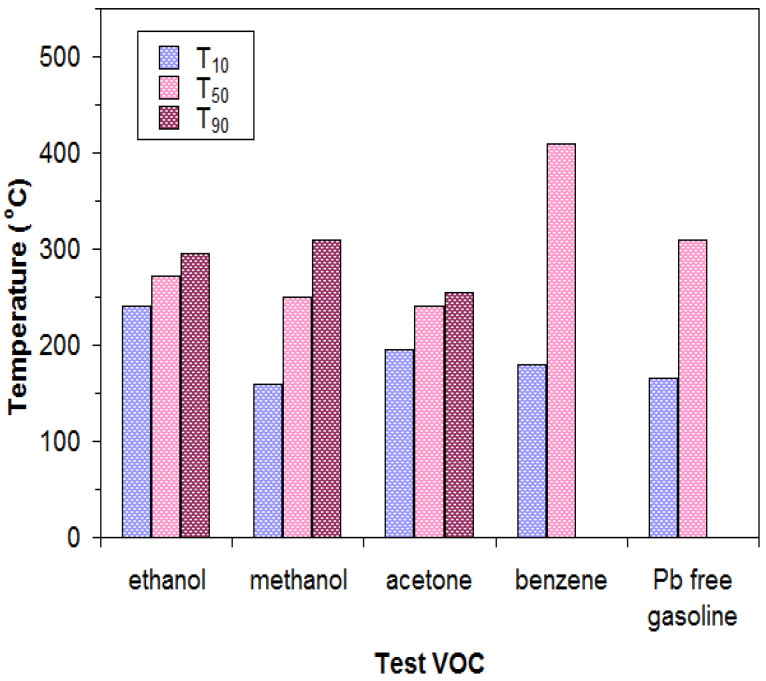
T_10_, T_50_, and T_90_ temperatures versus tested VOCs for the LaFeO_3_ catalyst.

Figure 6 presents a series of main indicators that can estimate the catalytic activity of the catalyst for the volatile organic compounds studied, namely (I) the temperature required for 10% gas conversion (T_10_), (II) the temperature required for 50% gas conversion (T_50_), and (III) the temperature required for 90% gas conversion (T_90_) [38,49].

The T_10_ temperatures for the volatile organic compounds studied are close to each other, namely 160 °C for methanol, 195 °C for acetone, 180 °C for benzene, and 165 °C for gasoline, except for ethanol, for which the T_10_ temperature has a value of 240 °C. At these temperatures, the catalytic reaction is started and is stable.

Regarding T_50_ temperatures, when the catalytic activity is sufficiently high and the interactions between the catalyst surface and the gas (reactants) are intense, their values are given in ascending order, taking into account that the lower the T_50_, the higher the catalytic activity of the catalyst for that gas. Thus, the T_50_ temperature has the lowest value for acetone (240 °C), followed by that for methanol (250 °C), ethanol (272 °C), Pb-free gasoline (310 °C), and benzene (410 °C). Taking into account the values of these temperatures, it can be stated that the catalytic activity of the studied lanthanum perovskite is higher in the case of alcohols and ketones compared to that of the studied hydrocarbons. Also, the T_90_ temperature values were determined for ethanol (295 °C), methanol (310 °C), and acetone (255 °C).

Taking these temperatures into account, it can be concluded that the catalyst based on lanthanum perovskite, LaFeO_3_, obtained by the preparation method used by us, seems to be the most active for acetone combustion; it can show a conversion of 90% at 255 °C and over 99% at 270 °C. The performance of the studied catalyst is remarkable; this can be compared to that of a catalyst based on Pt/Al_2_O_3_/Al, which could achieve a 95% conversion of acetone at 290 °C [50,51]. Also, this catalyst shows high performances also in terms of the conversion of the studied alcohols. For example, in the case of ethanol, it shows a conversion of 90% at 295 °C and over 99% at 310 °C, approaching that of a catalyst based on Au/γ-Al_2_O_3_, which could achieve a conversion of 99.6% of ethanol at a temperature of 290 °C [52]. In Table 1, the values of the temperatures T_10_, T_50_, T_90_, and T_99_ for a series of oxide catalysts containing Pt or Au in their structure for the combustion of acetone and ethanol vapors are presented for comparison.

The remarkable catalytic activity of this catalyst by achieving a very high conversion degree in the case of acetone and ethanol indicates the existence of reactive oxygen species on the surface of this catalyst (OH^−^/O_2_, O_2_^2−^/O^−^ si O^2−^), proven by XPS analyses (Figure 4c), that are weakly anchored and are much more available for the oxidation of acetone and ethanol compared to the other VOCs studied. Also, the behavior of sudden increase in the degree of conversion in a narrow temperature range (Figure 5) may be due to the rearrangement of the lattice structure and, consequently, of the structure of the active states on which the catalytic properties of this perovskite depend [8,38,39].

Table 2 shows the values of the degree of conversion of the studied VOCs at the temperatures of 270 °C and 550 °C, as well as the values of the kinetic parameters (reaction rate constant, *k*; and apparent activation energy, *E_app_*) for their oxidation by the LaFeO_3_ catalyst. The apparent activation energies of the LaFeO_3_ catalyst for the combustion reactions of ethanol, methanol, acetone, benzene, and unleaded gasoline were obtained by means of the Arrhenius-type plot of the ln*k* versus 1/*T* for low conversion regions (<12%) [5,8,39]. The Arrhenius equation is expressed as follows [39,56,57]:

*k* = A exp(E_app_/RT) (7)

where *k* is the reaction rate constant, *A* is a pre-exponential factor, *R* is the universal gas constant, *T* is the absolute temperature, and *E_app_* is the apparent activation energy.

The values of the apparent activation energies of the catalyst in the combustion reactions of ethanol, methanol, acetone, benzene, and Pb-free gasoline are in the range of 20.16–133.44 KJ/mol (Table 2). The highest value of the apparent activation energy is observed for ethanol (133.44 KJ/mol), considering the fact that the combustion reaction in this case begins at a rather high temperature (200 °C). The values obtained are comparable to those presented by other authors who have investigated a series of oxide compounds with various structures [8,34,39,58,59,60].

Specific catalytic activity can be characterized in terms of the reaction rate normalized to the specific surface area. The higher the reaction rate, the more pronounced is the catalytic activity that a catalyst can exhibit [38]. The highest reaction rate value (8.166 × 10^−2^ μmol s^−1^m^−2^) was obtained for the combustion of acetone by the LaFeO_3_ catalyst. It showed the best catalytic activity in the combustion of acetone vapor, reaching a conversion degree of over 99% at a temperature of 270 °C. The lowest reaction rate value (0.300 × 10^−2^ μmol s^−1^m^−2^) was obtained for the combustion of benzene vapors, the catalyst also presenting the weakest catalytic activity in the conversion of benzene (Figure 5).

The catalyst based on lanthanum perovskite, LaFeO_3_, obtained by the specified preparation method, can be recommended for the combustion of acetone, ethanol, and methanol vapors. The performance of this catalyst is remarkable and can be compared to that of a catalyst that includes noble metals in its structure.

## 4. Conclusions

This work presents the method of obtaining porous nanocrystalline powders of lanthanum perovskite, LaFeO_3_, by the sol–gel self-combustion method, using polyvinyl alcohol as colloidal medium. Also, this work presents the results of investigations of these powders from the point of view of catalytic combustion at moderate temperatures (50–550 °C) for a series of VOCs: ethanol, methanol, acetone, benzene, and Pb-free gasoline.

The catalytic activity of the studied catalyst is influenced to a greater extent by the reaction temperature in the case of alcohols and ketones compared to the case of hydrocarbons.

The lanthanum perovskite catalyst, LaFeO_3_, obtained by the presented preparation method, is the most active for acetone combustion; it shows a conversion degree value of 90% at 255 °C and over 99% at 270 °C. The conversion degree in the case of ethanol vapors is 90% at 295 °C and over 99% at 310 °C. This catalyst also shows high performance in terms of methanol vapors, where the conversion degree reaches a value of 97% at a temperature of 330 °C. The degree of conversion for Pb-free gasoline and benzene reaches somewhat lower values, over 88% at much higher temperatures, 470 °C and 550 °C, respectively.

The catalyst based on lanthanum perovskite, LaFeO_3_, obtained by the preparation method specified by us can be successfully recommended for the combustion of acetone, ethanol, and methanol vapors.

## Figures and Tables

**Table 1:** Conversion temperature values for catalysts containing Pt or Au for the combustion of acetone and ethanol.

Catalyst	VOC	T_10_ (°C)	T_50_ (°C)	T_90_ (°C)	T_99_ (°C)	References
LaFeO_3_	Acetone	195	240	255	270	This work
Pt/Al_2_O_3_	Acetone	–	222	246	259	[53]
Pt-Zr/Al_2_O_3_	Acetone	–	245	261	281	[53]
Pt-TiO_2_	Acetone	–	220	244	260	[53]
Pt-Zr/TiO_2_	Acetone	–	235	256	275	[53]
Pt/Al_2_O_3_/Al	Acetone	–	~208	~270	~341	[51]
0.11 wt% Pt/TiO_2_	Acetone	162	220	260	–	[54]
0.17 wt% Pt/TiO_2_	Acetone	143	205	245	–	[54]
0.46 wt% Pt/TiO_2_	Acetone	140	192	231	–	[54]
1.40 wt% Pt/TiO_2_	Acetone	135	180	208	–	[54]
LaFeO_3_	Ethanol	240	272	295	310	This work
1.5 wt% Au/γ-Al_2_O_3_	Ethanol	–	–	–	~290	[52]
2÷2.5 wt% Au/γ-Al_2_O_3_	Ethanol	–	–	–	~280	[52]
Pt/Al_2_O_3_	Ethanol	–	158	220	240	[55]

**Table 2:** The degree of conversion of the studied VOCs at 270 °C and at 550 °C and the kinetic parameters for the LaFeO_3_ catalyst.

VOCs/ LaFeO_3_	Conversion at 270 °C (%)	Conversion at 550 °C (%)	Reaction Rate * (μmol s^−1^ m^−2^)	Apparent Activation Energy ** (KJ/mol)
Ethanol	45.54	99.03	1.066 × 10^−2^	133.449
Methanol	64.54	97.54	1.818 × 10^−2^	20.166
Acetone	99.05	99.70	8.166 × 10^−2^	21.631
Benzene	15.76	88.50	0.300 × 10^−2^	22.238
Pb-free gasoline	30.80	89.70	0.645 × 10^−2^	22.237

* Reaction rate (at 270 °C) for VOC concentration at low conversion per unit surface area of catalyst. ** Apparent activation energy for low conversions regions.

## Data Availability

The raw data supporting the conclusions of this article will be made available by the authors on request.
